# Relationship between Retinal Thickness and Plasma Biomarkers in a Memory Centre Population

**DOI:** 10.1002/alz70856_103009

**Published:** 2025-12-26

**Authors:** Giulia Rugarli, Giordano Cecchetti, Roberto Santangelo, Giovanni Napoli, Federico Coraglia, Edoardo G. Spinelli, Francesca Caso, Giuseppe Magnani, Massimo Filippi, Federica Agosta

**Affiliations:** ^1^ Vita‐Salute San Raffaele University, Milan, Italy; ^2^ IRCCS San Raffaele Scientific Institute, Milan, Italy; ^3^ IRCCS San Raffaele Scientific Institute, Milan, MI, Italy; ^4^ University Vita‐Salute San Raffaele, Milan, MI, Italy; ^5^ Vita‐Salute San Raffaele University, Milan, MI, Italy

## Abstract

**Background:**

Alzheimer's disease (AD) is a neurodegenerative disorder characterized by cognitive decline and the accumulation of beta‐amyloid and tau proteins. Optical coherence tomography (OCT) is a non‐invasive imaging method that evaluates retinal layers, which may serve as biomarkers for AD. Plasma biomarkers, including beta‐amyloid ratios, phosphorylated tau (*p*‐tau), and neurofilament light chain (NfL), provide minimally invasive diagnostic and monitoring tools. This study evaluates how retinal thickness changes in relation to different plasma biomarkers.

**Method:**

This cross‐sectional study included 145 consecutive patients with cognitive disturbances due to heterogeneous conditions from the tertiary Memory Clinic at San Raffaele Hospital, Milan, Italy. They all underwent OCT imaging and blood sampling for plasma biomarker assessment. Linear regression analyses were performed to assess associations between the average retinal layer thicknesses of both eyes and plasma biomarkers, adjusting for age, sex, and disease duration. Patients were stratified into three groups based on dual pTau217 cutoffs, calculated on a sample of 184 individuals to achieve 97% sensitivity and specificity for identifying pathological CSF pTau181/Aβ42 ratios: Group 1 (p217 < 0.1325; no amyloid pathology), Group 2 (0.1325 ≤ p217 < 0.356; gray zone), and Group 3 (p217 ≥ 0.356; amyloid pathology). Subgroup‐specific regressions were performed, and intergroup comparisons were analyzed.

**Result:**

A positive correlation was identified between the temporal retinal nerve fiber layer (T_RNFL) and the beta‐amyloid 42/40 ratio (Ab42/40) in the overall cohort. In Group 1, a negative correlation was found between the inferior RNFL (I_RNFL) and Ab42/40. Group 2 exhibited a positive correlation between I_RNFL and NfL levels. In Group 3, negative correlations were observed between plasma phosphorylated tau 181 (p181) and the global RNFL (G_RNFL), superior RNFL (S_RNFL), and global ganglion cell and inner plexiform layers (GCL‐IPL). No significant differences were observed in intergroup comparisons.

**Conclusion:**

This study highlights the potential of OCT‐derived retinal measurements as complementary biomarkers for AD. The findings suggest complex, stage‐specific associations between retinal thickness and plasma biomarkers, underscoring the importance of stratifying patients by amyloid status. These results contribute to understanding neurodegenerative processes and may inform diagnostic and monitoring strategies for AD.

**Funding**: Fujirebio Italia

